# Case Report: Robotic-assisted resection of intra-abdominal aggressive fibromatosis and Boari flap ureteroneocystostomy for hydronephrosis

**DOI:** 10.3389/fonc.2026.1823829

**Published:** 2026-05-15

**Authors:** Lei Yang, Yichuan Wang, Han Hao, Kunlin Yang, Wei Yu

**Affiliations:** 1 Department of Urology, Peking University First Hospital, Peking University, Beijing, China; 2 Institute of Urology, Peking University, Beijing, China; 3 National Urological Cancer Center, Beijing, China

**Keywords:** aggressive fibromatosis, Boari flap, desmoid-type fibromatosis, hydronephrosis, robotic-assisted surgery, ureteral obstruction, ureteral reconstruction

## Abstract

**Background:**

Aggressive fibromatosis (AF), also known as desmoid-type fibromatosis, is a rare, locally infiltrative soft-tissue tumor with no metastatic potential but a significant risk of local recurrence. Intra-abdominal AF may encase adjacent organs and vascular structures, occasionally leading to organ dysfunction. Ureteral involvement resulting in hydronephrosis is uncommon and presents complex diagnostic and therapeutic challenges, particularly when reconstruction of the urinary tract is required.

**Case summary:**

We report a 28-year-old male patient in whom right-sided hydronephrosis was incidentally detected during routine examination. Imaging revealed a 6 cm pelvic mass encasing the distal ureter and displacing the iliac vessels. Biopsy suggested a low-grade spindle-cell tumor. Due to progressive ureteral obstruction, surgical intervention was indicated. The patient underwent robotic-assisted laparoscopic tumor resection with segmental ileal resection and intracorporeal anastomosis. Distal ureteral involvement necessitated resection and reconstruction using a 7-cm Boari bladder flap ureteroneocystostomy over a double-J stent. Histopathological examination confirmed aggressive fibromatosis. Postoperative recovery was uneventful. At three-month follow-up, imaging demonstrated resolution of hydronephrosis and no evidence of recurrence.

**Conclusions:**

Intra-abdominal aggressive fibromatosis with ureteral encasement is rare and may require simultaneous oncologic resection and complex urinary tract reconstruction. A single-stage robotic-assisted approach can facilitate precise tumor excision and tension-free ureteral reconstruction while preserving renal function. Individualized surgical planning is essential in managing organ-compromising desmoid tumors.

## Introduction

Aggressive fibromatosis (AF), also known as desmoid-type fibromatosis, is a rare soft-tissue tumor arising from fibroblastic or myofibroblastic proliferation. Although it lacks metastatic potential, AF is characterized by locally infiltrative growth and a high propensity for local recurrence (1, 2). The estimated incidence is approximately 2–4 cases per million population annually, accounting for less than 0.03% of all tumors (2).

AF can be classified into extra-abdominal, abdominal wall, and intra-abdominal types (3). Intra-abdominal AF most commonly arises from the mesentery or retroperitoneum and poses unique management challenges due to its proximity to major vascular structures, bowel loops, ureters, and pelvic organs (4, 5). Unlike superficial lesions, intra-abdominal tumors may remain asymptomatic until they compress or encase adjacent structures, leading to secondary organ dysfunction.

Ureteral involvement by intra-abdominal AF is uncommon but clinically significant. Persistent ureteral obstruction can result in progressive hydronephrosis, renal function deterioration, and long-term morbidity. Only isolated case reports describe AF presenting primarily with hydronephrosis (6, 7). The rarity of this presentation complicates the establishment of standardized management strategies.

Over the past decade, the therapeutic paradigm for AF has shifted from routine surgical excision to a more conservative “watchful waiting” approach, particularly for asymptomatic or stable lesions (8, 9). International consensus guidelines recommend active surveillance as first-line management in many cases (9). However, intervention is indicated in patients with progressive disease, refractory symptoms, or organ compromise (5, 9).

When ureteral encasement and hydronephrosis are present, management must simultaneously address oncologic control and preservation of renal function. Surgical resection in the pelvis is technically demanding, particularly when the tumor is adjacent to iliac vessels and involves bowel or distal ureter. Reconstruction may require bowel resection and complex urinary tract reconstruction, such as Boari flap ureteroneocystostomy, to achieve tension-free restoration of urinary continuity (10, 11).

Minimally invasive approaches, including robotic-assisted surgery, have expanded reconstructive possibilities in confined anatomical spaces (12, 13). The enhanced visualization and instrument dexterity of robotic systems may facilitate precise dissection and intracorporeal reconstruction in selected complex pelvic cases (10, 13). Nevertheless, evidence regarding robotic management of intra-abdominal AF with ureteral involvement remains limited.

To our knowledge, few reports describe a single-stage robotic-assisted resection of intra-abdominal aggressive fibromatosis combined with bowel resection and Boari flap ureteral reconstruction for hydronephrosis. Herein, we present a rare case of distal ureteral encasement by intra-abdominal AF successfully managed with organ-preserving robotic surgery.

## Case introduction

**Figure 1:**
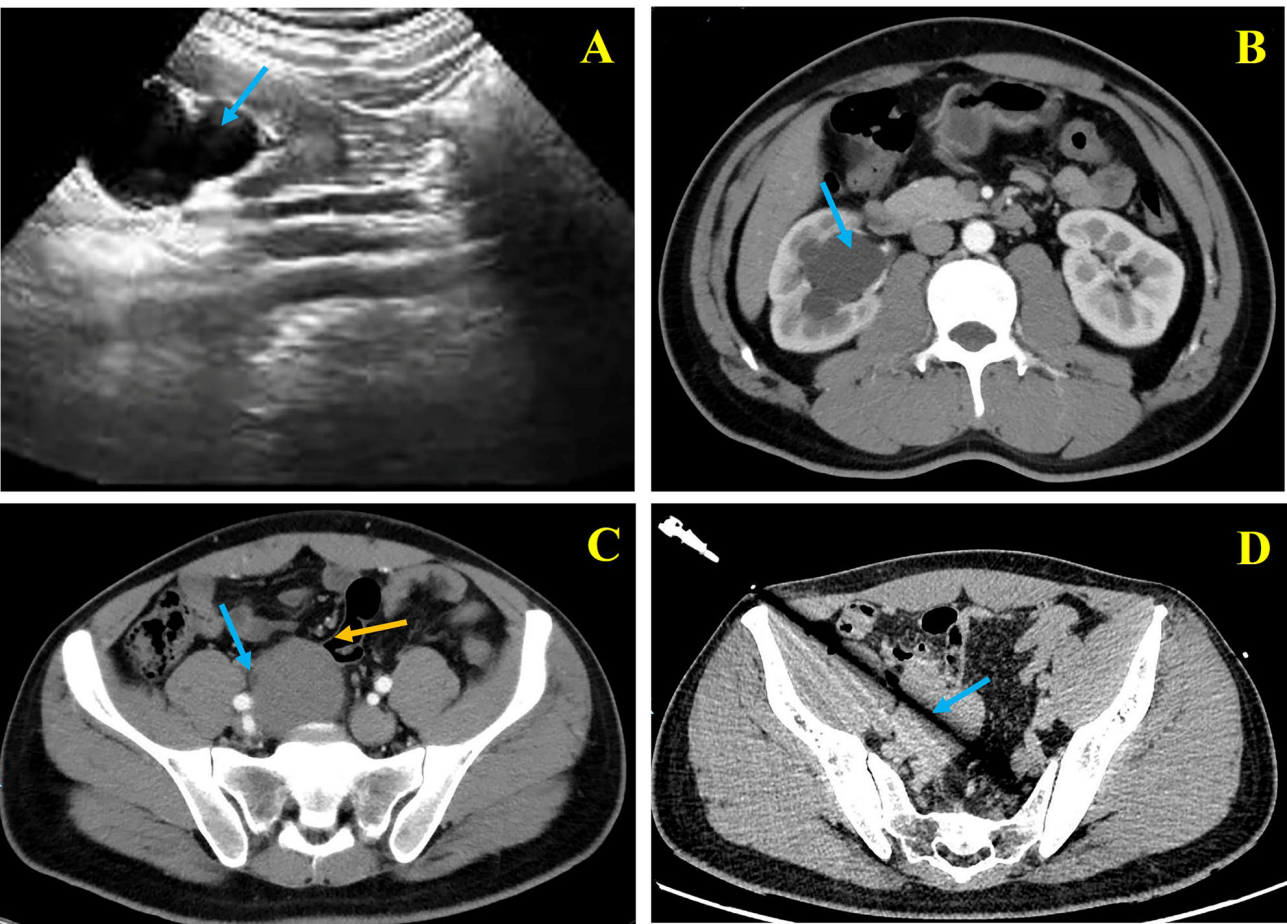
Preoperative imaging of the patient. **(A)** (ultrasound) and **(B)** (CT) showing right hydronephrosis (blue arrow). **(C)** Contrast-enhanced CT demonstrating right pelvic mass encasing distal ureter (blue arrow) with indistinct margins with adjacent small bowel (yellow arrow). **(D)** CT-guided core biopsy in tumor (blue arrow).

A 28-year-old male with no significant past medical history was referred to our department after right-sided hydronephrosis was incidentally detected during a routine health examination. The patient denied flank pain, hematuria, dysuria, urinary frequency, weight loss, or constitutional symptoms. Physical examination was unremarkable, and no palpable abdominal mass was identified. Laboratory evaluation, including serum creatinine and urinalysis, was within normal limits. Ultrasonography demonstrated right renal pelvic dilatation and proximal ureteral expansion. Additionally, a hypoechoic pelvic mass adjacent to the right iliac vessels was identified (**Figure 1A**). Contrast-enhanced computed tomography (CT) revealed a 5.4 × 6.1 × 6.0 cm well-defined, non-encapsulated soft-tissue mass located in the right pelvis. The lesion was isodense to skeletal muscle on non-contrast images and exhibited progressive mild-to-moderate enhancement. The tumor displaced the right external iliac vessels and encased the distal ureter, resulting in marked upstream hydroureteronephrosis (**Figures 1B, C**). No enlarged lymph nodes or distant metastases were observed. CT-guided core needle biopsy demonstrated spindle-shaped cells arranged in fascicles within a collagenous stroma, without significant atypia or necrosis, suggestive of a low-grade spindle-cell neoplasm (**Figure 1D**). Cystoscopic retrograde pyelography confirmed distal ureteral narrowing. Because retrograde stent placement failed and the patient already had marked hydroureteronephrosis, further delay carried a risk of irreversible renal functional loss. Considering the patient’s young age and the localized, potentially resectable nature of the lesion, the multidisciplinary team, including urology, general surgery, radiology, and pathology specialists, favored definitive single-stage surgical management rather than initial systemic therapy or temporary nephrostomy alone.

Intra-abdominal aggressive fibromatosis (AF) is characterized by locally infiltrative growth and a variable natural history (1, 14). Current consensus guidelines advocate active surveillance for asymptomatic or stable disease; however, intervention is indicated in cases with progressive symptoms or organ compromise (5, 9). In this case, persistent ureteral obstruction with hydronephrosis constituted a clear indication for operative management. The primary surgical objectives were: complete macroscopic tumor excision, preservation of adjacent vascular structures, restoration of urinary tract continuity, and maintenance of renal function. Open surgery was considered; however, given the deep pelvic location and anticipated need for both bowel resection and ureteral reconstruction, a minimally invasive robotic-assisted approach was selected. The robotic platform allows improved visualization and dexterity within confined pelvic spaces, which is particularly relevant when operating near major vessels and performing complex intracorporeal suturing (12, 13). Alternative options included initial nephrostomy diversion or systemic therapy; however, given the localized nature of the tumor and absence of metastatic disease, definitive surgical management was preferred.

**Figure 2:**
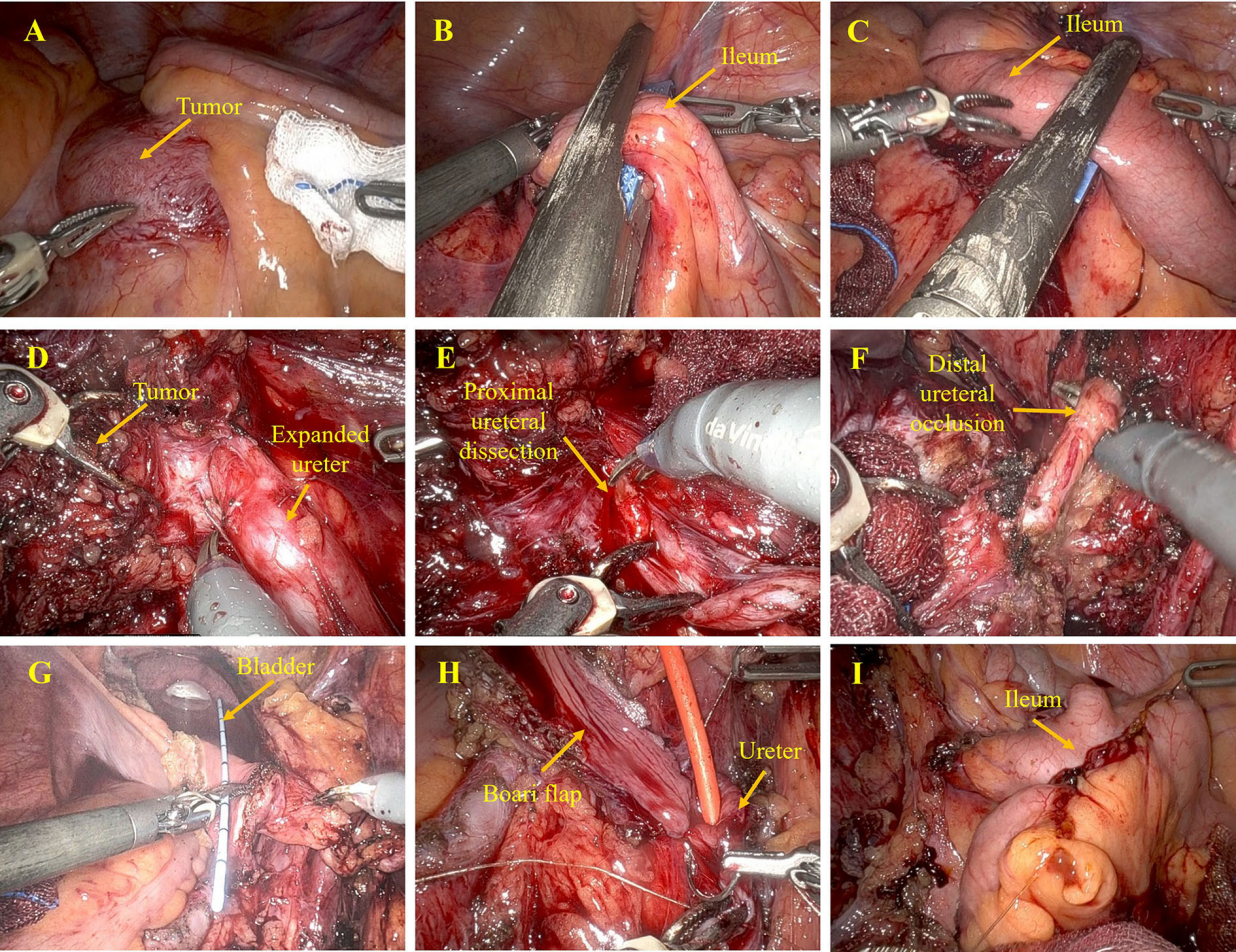
The procedure of surgery. **(A)** Tumor involving terminal ileum. **(B, C)** showing stapled ileal transection. **(D)** right ureter dissection. **(E)** Ureteral division at stenosis. **(F)** Distal ureter occlusion. **(G)** Bladder flap creation. **(H)** Ureter-to-bladder-flap anastomosis. **(I)** Ileal anastomosis.

Under general anesthesia, the patient was positioned supine. The procedure was performed using the da Vinci Xi surgical system (Intuitive Surgical, Sunnyvale, CA, USA). Intraoperatively, dense adhesions were observed between the tumor and the terminal ileum (**Figure 2A**). Careful sharp and blunt dissection was performed to mobilize the mass from the pelvic sidewall and iliac vessels without vascular injury. Due to serosal invasion of approximately 10 cm of terminal ileum, segmental ileal resection was performed (**Figures 2B, C**). A side-to-side stapled ileoileal anastomosis was constructed intracorporeally (**Figure 2I**). The robotic system facilitated precise dissection in close proximity to the iliac vessels and allowed controlled mobilization of the tumor.

The right ureter was identified proximally and dissected distally to the level of tumor encasement (**Figure 2D**). The stenotic segment was transected, with immediate urinary efflux confirming proximal patency (**Figures 2E, F**). After complete tumor excision, reconstruction was performed using a 7-cm Boari bladder flap. The bladder was mobilized, and a flap was created, tubularized, and anastomosed to the spatulated proximal ureter over a double-J stent using interrupted absorbable sutures (**Figures 2G, H**). Boari flap ureteroneocystostomy is a well-established technique for distal ureteral defects when tension-free reimplantation is not feasible (10, 11, 15). In this case, the length of ureteral involvement precluded simple reimplantation, making Boari reconstruction necessary. Intraoperative leak testing confirmed watertight anastomosis. A pelvic drain was placed.

**Figure 3:**
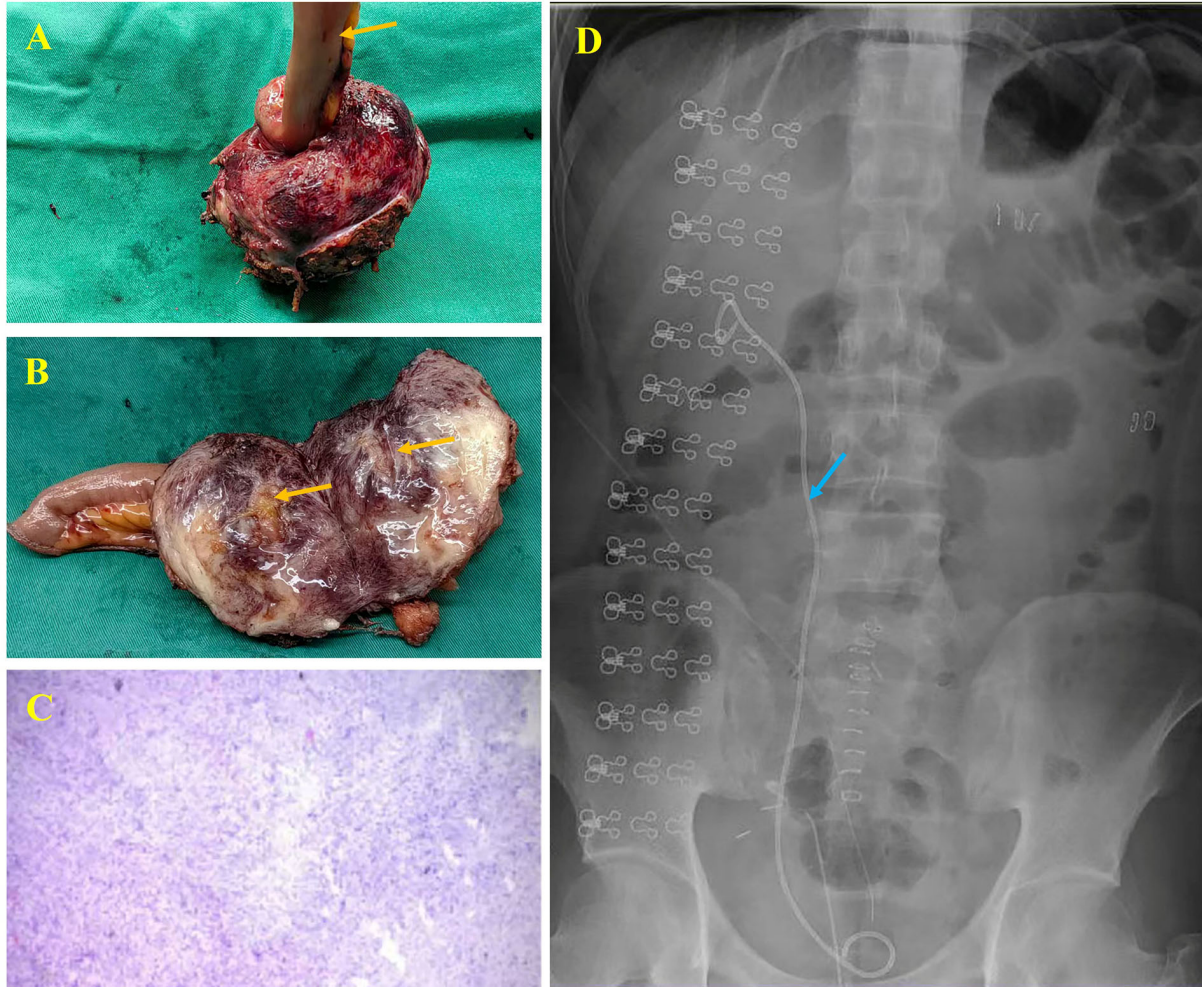
Postoperative data of the patient. **(A, B)** Gross specimen with ileal involvement (yellow arrow). **(C)** HE staining showing the fibromatosis. **(D)** postoperative KUB confirming right double-J stent position (blue arrow).

Gross examination revealed a firm, slate-blue mass with a whorled cut surface, partially enveloping the ileum (**Figures 3A, B**). Microscopically, the tumor consisted of uniform spindle cells arranged in sweeping fascicles within a collagenous stroma. There was infiltration into adjacent tissue without significant atypia, mitotic activity, or necrosis (**Figure 3C**). Immunohistochemistry demonstrated nuclear β-catenin positivity and smooth muscle actin (SMA) positivity. CD34 staining was positive in vascular structures. The tumor was negative for desmin, caldesmon, and S-100 protein. Ki-67 proliferation index was below 3%. These findings confirmed the diagnosis of aggressive fibromatosis. Nuclear β-catenin expression is a characteristic feature of sporadic desmoid-type fibromatosis and supports diagnostic accuracy (16, 17).

The postoperative course was uneventful. The patient resumed oral intake on postoperative day 2 and was discharged on postoperative day 6. Postoperative kidney-ureter-bladder radiography confirmed correct positioning of the double-J stent (**Figure 3D**). The stent was removed three weeks after surgery. Adjuvant therapy with oral celecoxib was initiated, given reports suggesting a potential role of NSAIDs in selected AF cases (18, 19). However, the role of NSAIDs in AF remains controversial and should not be interpreted as standard disease-modifying therapy. Indeed, non-operative options include temporary urinary diversion and contemporary systemic therapies when active treatment is required. Historically used agents such as NSAIDs or anti-hormonal therapy have limited and controversial evidence and would not be expected to provide prompt relief of fixed mechanical ureteral obstruction in the present case. At three-month follow-up, CT urography demonstrated complete resolution of hydronephrosis and preserved renal function. No evidence of tumor recurrence was observed. Long-term imaging surveillance was scheduled, given the known risk of local recurrence associated with aggressive fibromatosis (14, 20). The patient has continued follow-up every 3 months at his local hospital. At the most recent evaluation, nearly 1 year after surgery, no radiologic evidence of recurrence was detected, and renal function remained preserved (serum creatinine 82.0 μmol/L; eGFR 112.5 mL/min/1.73 mL/.

## Discussion

AF involving the distal ureter represents a rare and technically demanding clinical scenario (6, 7). Although AF lacks metastatic potential, its infiltrative growth pattern and tendency to encase adjacent structures can result in significant morbidity (1, 5). In the present case, tumor involvement of the distal ureter with secondary hydronephrosis created a composite problem that required oncologic control, preservation of major pelvic vessels, bowel management, and restoration of urinary tract continuity within a confined anatomical space.

The pelvis contains a dense concentration of vital structures, including the iliac vessels, ureter, bowel loops, autonomic nerves, and bladder. Intra-abdominal AF most frequently arises from the mesentery or retroperitoneum and may extend to adjacent organs through fascial planes rather than discrete invasion (4, 5). This growth pattern often results in circumferential encasement of structures such as the ureter rather than focal obstruction. Ureteral involvement is uncommon but clinically significant. Prolonged obstruction can lead to progressive hydronephrosis, renal function deterioration, and ultimately irreversible renal damage if not addressed. Reports of AF presenting primarily as ureteral obstruction remain scarce in the literature (6, 7). From a reconstructive standpoint, distal ureteral defects pose unique challenges. Direct ureteroneocystostomy may be feasible for short segments; however, when longer resections are required, tension-free reconstruction must be ensured to prevent stricture formation and anastomotic failure (10, 11, 15). In this case, the extent of ureteral encasement precluded simple reimplantation, necessitating Boari flap reconstruction to bridge the defect while preserving renal function. A Boari flap was preferred because the defect was distal and could be bridged with a tension-free bladder-based reconstruction after bladder mobilization. Compared with ileal ureter substitution, this approach preserved native urothelial tissue and avoided additional bowel interposition and its attendant gastrointestinal and metabolic morbidity, an important consideration in a young patient who already required segmental ileal resection. Renal autotransplantation was not favored because it would have introduced greater operative and vascular complexity without a clear reconstructive advantage for a distal ureteral defect that was amenable to Boari flap reconstruction.

The management of aggressive fibromatosis has evolved significantly over the past decade. Current international consensus guidelines recommend active surveillance as first-line management for asymptomatic or stable disease, given the unpredictable natural history and potential for spontaneous stabilization (9). However, intervention is indicated in cases of progressive disease, refractory pain, or organ compromise. In the present patient, persistent ureteral obstruction with hydronephrosis constituted a clear indication for active treatment. Non-operative alternatives such as systemic therapy (e.g., NSAIDs, anti-estrogen agents, tyrosine kinase inhibitors) may achieve tumor stabilization but are unlikely to provide rapid relief of mechanical obstruction (16, 18, 19). Temporary urinary diversion via percutaneous nephrostomy could have been considered. Nevertheless, given the localized nature of the lesion and the patient’s young age, definitive surgical excision with immediate reconstruction was favored to achieve both oncologic control and durable functional restoration. Open surgery has traditionally been the standard approach for complex pelvic AF (14). While it provides wide exposure, it is associated with increased postoperative pain, longer recovery, and higher morbidity. Conventional laparoscopy offers a minimally invasive alternative but may be technically limited in narrow pelvic spaces, particularly when meticulous vascular dissection and complex intracorporeal suturing are required.

Robotic-assisted surgery has expanded the possibilities of minimally invasive management for complex pelvic pathology (12, 13). The system provides three-dimensional magnified visualization and articulated instruments with enhanced dexterity, facilitating precise dissection around critical structures such as the iliac vessels and ureter. In this case, robotic assistance enabled careful separation of the tumor from major vessels and safe mobilization of the ureter in a confined anatomical field (10, 15). Moreover, the platform facilitated intracorporeal bowel anastomosis and construction of a Boari flap ureteroneocystostomy with precise suturing. Although evidence remains limited regarding robotic management of intra-abdominal AF, robotic approaches have been successfully applied in other complex desmoid tumor resections and reconstructive procedures (12). Nevertheless, the advantages of robotic surgery must be balanced against factors such as cost, equipment availability, and surgeon experience.

Aggressive fibromatosis is characterized by a high local recurrence rate, reported between 20% and 40% depending on margin status and tumor biology (8, 14, 20). Complete macroscopic resection remains an important goal in symptomatic cases; however, margin-negative resection does not eliminate recurrence risk. Immunohistochemical demonstration of nuclear β-catenin supports the diagnosis of sporadic AF and reflects underlying CTNNB1 mutations, which may influence recurrence patterns (16, 17). Given the known risk of local relapse, structured imaging surveillance is mandatory (9). At one-year follow-up, our patient demonstrated complete resolution of hydronephrosis and preserved renal function without radiological evidence of recurrence. Long-term follow-up will be essential to assess durability. Failure to adequately relieve ureteral obstruction could result in progressive renal dysfunction, recurrent infections, and long-term impairment of quality of life. In the present case, simultaneous oncologic clearance and urinary tract reconstruction allowed preservation of renal function and rapid postoperative recovery. Otherwise, the absence of preoperative MRI is a limitation of this report. Nevertheless, CT remains a practical and acceptable initial imaging modality for intra-abdominal desmoid tumors, particularly in urgent obstructive presentations, while histologic confirmation remains essential for definitive diagnosis.

## Conclusion

Robotic-assisted tumor resection combined with intracorporeal bowel management and Boari flap ureteroneocystostomy represents a safe and effective single-stage approach for the treatment of intra-abdominal aggressive fibromatosis complicated by distal ureteral obstruction. This strategy allows complete macroscopic tumor removal while simultaneously restoring urinary tract continuity and preserving renal function. In cases of ureteral encasement, simple ureteral reimplantation may not provide a tension-free reconstruction. The use of a Boari flap enables adequate bridging of longer distal ureteral defects and ensures durable urinary drainage. Furthermore, the minimally invasive robotic platform facilitates precise dissection in anatomically confined pelvic spaces and supports meticulous intracorporeal suturing during reconstructive steps. Although open surgery remains a valid and widely practiced option for complex pelvic desmoid tumors, the integration of robotic-assisted resection with immediate urinary tract reconstruction in a single procedure may reduce surgical trauma and promote faster recovery in selected patients. Importantly, should recurrence occur, additional systemic therapy or further surgical intervention remains feasible. While robotic techniques and ureteral reconstruction methods have each been described separately in the management of pelvic tumors, reports combining robotic tumor excision, bowel resection, and Boari flap reconstruction for intra-abdominal aggressive fibromatosis are exceedingly limited. In contrast to staged diversion followed by delayed reconstruction, our approach achieved oncologic control and functional restoration simultaneously. Short-term follow-up in this case confirms resolution of hydronephrosis, preservation of renal function, and absence of early recurrence. Long-term surveillance remains essential given the known recurrence risk of aggressive fibromatosis. Overall, this case highlights the feasibility of a tailored, organ-preserving surgical strategy in the management of rare intra-abdominal desmoid tumors with ureteral involvement.

## Data Availability

The raw data supporting the conclusions of this article will be made available by the authors, without undue reservation.
